# Severe Necrotizing Community‐Acquired Pneumonia and Bilateral Empyema in an Immunocompetent Patient due to *Fusobacterium necrophorum*

**DOI:** 10.1155/crpu/5505327

**Published:** 2026-01-14

**Authors:** Deborah Shefa, Aryan Shiari, Marina Antic, Buddhi P. Hatharaliyadda, Juan Vera Gomez, David Ladin, Igor Dumic

**Affiliations:** ^1^ Department of Hospital Medicine, Mayo Clinic Health System, Eau Claire, Wisconsin, USA, mayoclinichealthsystem.org; ^2^ Department of Pulmonary Medicine and Critical Care, Mayo Clinic Health System, Eau Claire, Wisconsin, USA, mayoclinichealthsystem.org; ^3^ Department of Infectious Disease, Mayo Clinic Health System, Eau Claire, Wisconsin, USA, mayoclinichealthsystem.org

**Keywords:** empyema, *Fusobacterium necrophorum*, incomplete Lemierre′s syndrome, necrotizing pneumonia

## Abstract

Necrotizing pneumonia and empyema caused by *Fusobacterium necrophorum* are uncommon. The classical presentation of Lemierre′s syndrome, characterized by pharyngotonsillitis and internal jugular vein thrombosis, is not always observed, and incomplete and atypical variants can cause diagnostic uncertainty and delay in treatment. We describe the case of a previously healthy 22‐year‐old male athlete who developed severe necrotizing community‐acquired pneumonia, bilateral empyema, and acute hypoxemic respiratory failure due to *F. necrophorum* infection, without the typical vascular thrombosis associated with Lemierre′s syndrome. Blood cultures and broad‐range PCR analysis of the empyema fluid confirmed the presence of *F. necrophorum*. The patient was initially treated with piperacillin–tazobactam, intravenous corticosteroids, and bilateral chest tube placement for empyema management. Treatment with piperacillin–tazobactam was complicated by drug‐induced liver injury requiring antimicrobial change to ampicillin–sulbactam. The patient was discharged on oral amoxicillin–clavulanic acid and completed a total of 5 weeks of antimicrobial therapy with complete recovery. This case highlights the importance of considering *F. necrophorum* as a potential pathogen in necrotizing community‐acquired pneumonia, even in immunocompetent adults, and in the absence of classic Lemierre′s syndrome features. Furthermore, its novelty and contribution to the medical literature stem from the patient′s presentation with bilateral empyema requiring bilateral drains, the combined blood culture and pleural fluid 16S confirmation of *F. necrophorum* infection, and the preceding steroid exposure that contributed to clinical deterioration.

## 1. Introduction

*Fusobacterium necrophorum* is a Gram‐negative, obligate anaerobic bacterium that is considered part of the normal human microbiota within the mucosal surfaces of the oropharynx, gastrointestinal, and female genital tract [1]. Despite its role as a commensal organism, *F. necrophorum* possesses pathogenic potential, particularly in adolescents and young adults, where it can act as a primary pathogen and cause invasive infections [2]. In individuals aged 13–40 years, *F. necrophorum* may represent the second most common bacterial cause of pharyngotonsillitis after *Streptococcus pyogenes* [1–3]. Recognized risk factors for the development of invasive infection include recent or concurrent pharyngotonsillitis and age between 13 and 30 years. Infectious mononucleosis and possible inherited thrombophilia may predispose individuals to invasive disease by facilitating progression to Lemierre′s syndrome (LS)—a potentially life‐threatening condition characterized by septic thrombophlebitis of the internal jugular vein following or during an oropharyngeal infection [1–3]. LS is often complicated by septicemia and the development of metastatic abscesses, particularly in the lungs and other organs [1–3]. The clinical presentation of *F. necrophorum* pharyngitis closely resembles that of streptococcal pharyngitis; however, it is associated with a higher risk of complications, including peritonsillar abscess formation and progression to LS [4, 5].

Necrotizing pneumonia is a severe form of pneumonia, characterized by liquefaction and necrosis of the lung parenchyma, leading to cavitation within areas of consolidation. This condition arises from tissue destruction caused by infection, most commonly due to highly virulent or toxin‐producing bacteria such as *Streptococcus pneumoniae* or *Staphylococcus aureus*, including strains that produce Panton–Valentine leukocidin (PVL) [6–8]. Radiologically, necrotizing pneumonia is identified on chest computed tomography (CT) by the presence of multiple thin‐walled cavities within consolidated lung regions [6–8]. In pediatric populations, necrotizing pneumonia more frequently affects previously healthy children. A recent history of influenza‐like illness is a strong risk factor for PVL‐positive *S. aureus* necrotizing pneumonia, particularly in young and otherwise healthy individuals [9–11]. Other common pathogens associated with community‐acquired necrotizing pneumonia include Gram‐negative bacilli such as *Klebsiella pneumoniae* and *Pseudomonas aeruginosa* [12, 13]. The mortality rate of necrotizing pneumonia in adults approaches 25%, with significantly higher mortality observed in patients requiring mechanical ventilation [7]. In this subgroup, the odds ratio for death is 27.6 [7]. Although *F. necrophorum* has been reported as a causative agent of necrotizing pneumonia and lung abscess, it remains an exceedingly rare cause of severe necrotizing pneumonia and sepsis in the absence of LS [14, 15].

## 2. Case Presentation

We report on a 22‐year‐old healthy athletic man without comorbidities who presented to urgent care complaining of 2 days of sore throat, chills, and vomiting. His physical exam was significant for oropharyngeal erythema and cervical lymphadenopathy. Vital signs were otherwise within normal limits. Rapid antigen testing for Group A *Streptococcus* and Monospot testing for infectious mononucleosis were both negative. A presumable diagnosis of viral pharyngitis was established, and the patient was prescribed a 3‐day course of prednisone (20 mg twice daily). The patient′s symptoms progressed as he failed to improve with symptomatic treatment and steroids, so 3 days later, he presented to the emergency department (ED) with progressive pleuritic left chest pain, palpitations, dyspnea, myalgias, and generalized weakness. The patient denied any aspiration events during episodes of vomiting. Social history was remarkable for stable housing; he was a college student and was not taking any illicit drugs, tobacco, or alcohol. The patient denied any recent travel outside Wisconsin, exposure to sick contacts, or any tick bites. He did not have any pets and was not sexually active. In the ED, he was afebrile (36.5), with sinus tachycardia (130–140 beats per minute), tachypnea (25–30 breaths per minute) with blood pressure of 137/95 mmHg. The physical exam was notable for an ill‐appearing man in mild distress with rapid and shallow breathing, using accessory muscles. Lungs were clear to auscultation, and oxygen saturation on room air was 88%. The oropharyngeal exam was unchanged compared to the exam from 3 days prior, with oropharyngeal erythema and cervical lymphadenopathy.

**Figure 2:**
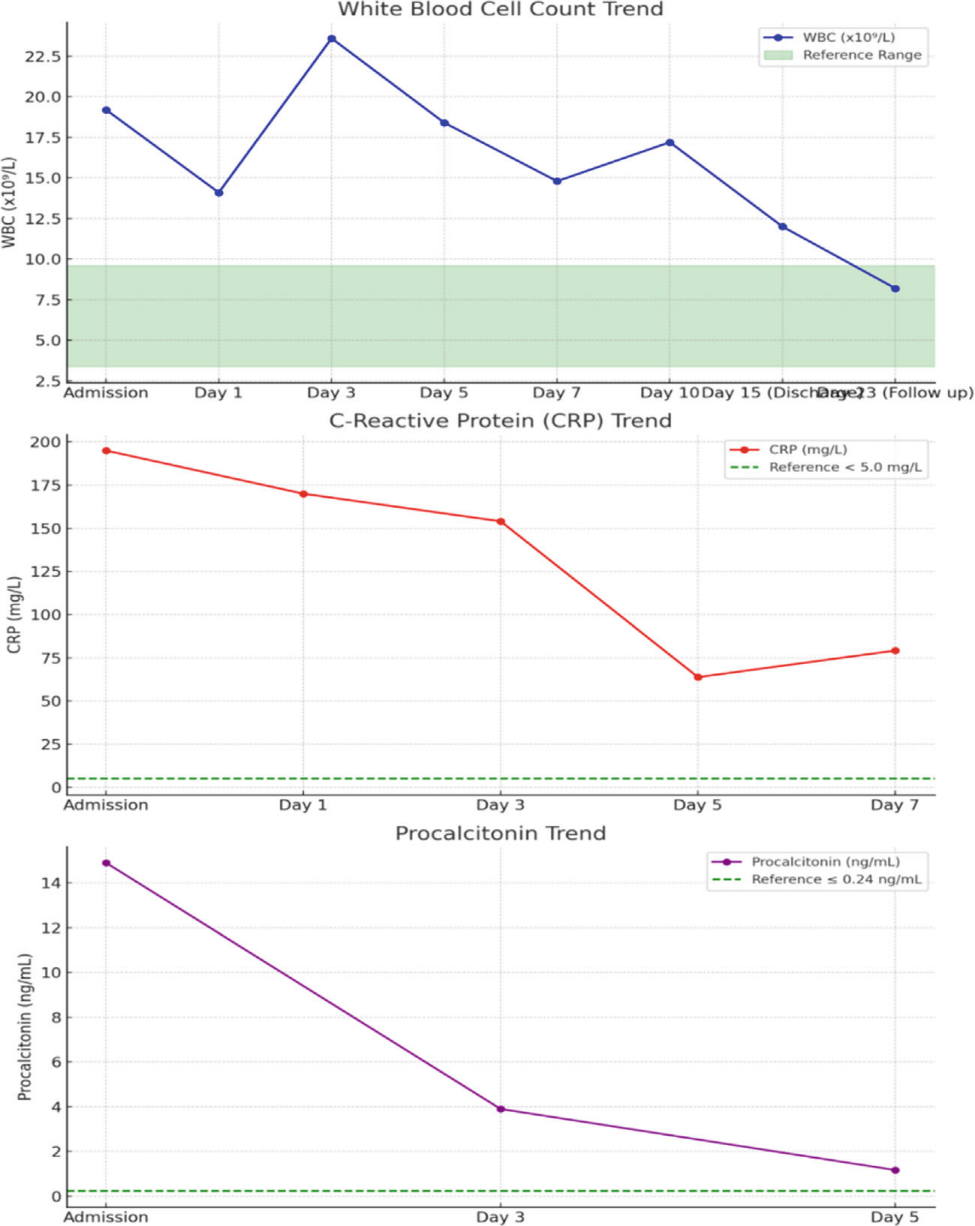
This line graph trends inflammatory markers including white blood cell count, C‐reactive protein, and procalcitonin.

Initial CT angiogram of the chest demonstrated patchy and ground‐glass opacities in both lung bases and development of cavitary lesions, suggestive of left‐sided necrotizing pneumonia (Figure 1). Transthoracic echocardiogram showed an ejection fraction of 60% and no wall motion abnormalities. Electrocardiogram (EKG) demonstrated sinus tachycardia, with normal intervals and without any ischemic changes. Initial and subsequent laboratory tests are shown in Table 1 and Figure 2.

Figure 1Contrast‐enhanced computed tomography (CT) pulmonary angiogram of the chest in the (a) sagittal, (b) axial, and (c) coronal planes. Imaging demonstrates bilateral basal patchy and ground‐glass opacities with early cavitary transformation (arrows), more prominent in the left lower lobe. These findings are consistent with necrotizing pneumonia.

**Figure:**
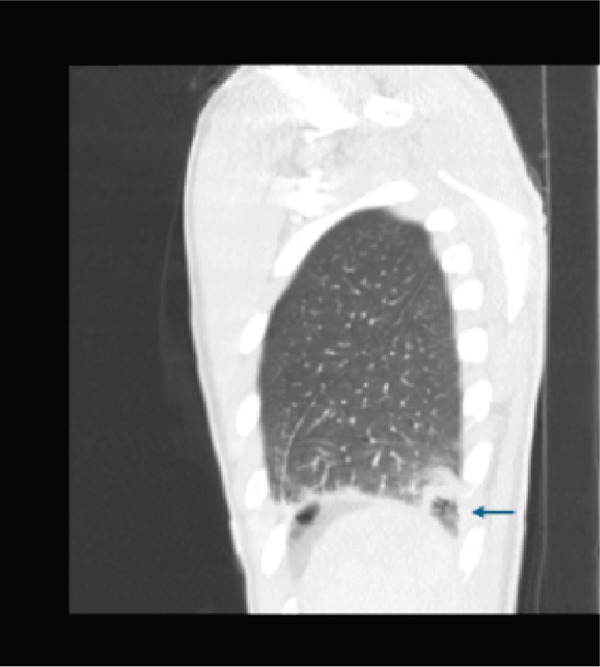
(a)

**Figure:**
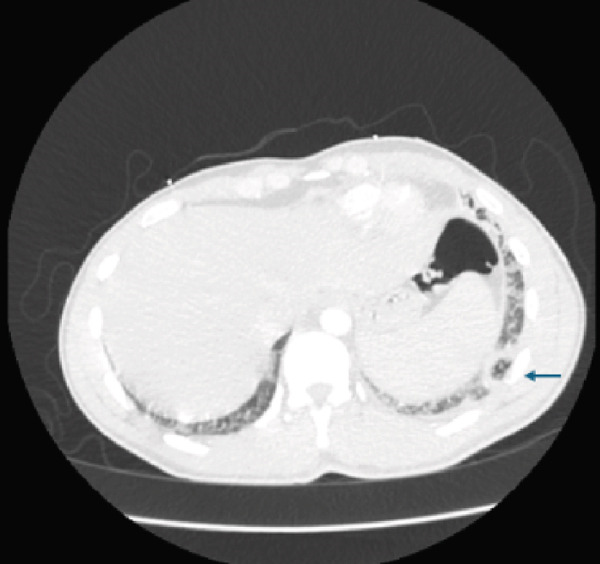
(b)

**Figure:**
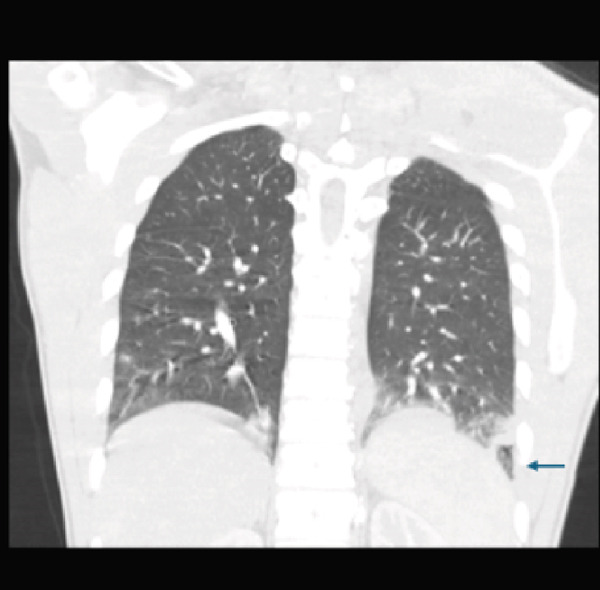
(c)

**Table 1:** This table illustrates a comprehensive work‐up for determining the etiology of necrotizing community‐acquired pneumonia in our case.

**Test**	**Blood specimen**	**Plural specimen**	**Urine specimen**	**Sputum specimen**	**Respiratory swab**
Cultures	Positive for *F. necrophorum*	Negative	—	—	—
*Histoplasma* and *Blastomyces* antigen (Ag) enzyme immunoassay	—	—	Negative		
*Legionella* Ag	—		Negative	—	—
*Streptococcus pneumoniae* Ag	—		Negative	—	Negative
Drug screen	—	—	Negative	—	—
Antinuclear antibody (Ab) HEp‐2 substrate	IgG negative	—	—	—	—
Anti‐neutrophil cytoplasmic Ab	Negative	—	—	—	—
Lyme Ab modified 2‐tier w/reflex	Negative	—	—	—	—
Infectious mononucleosis, rapid test	Negative	—	—	—	—
*Cytomegalovirus* DNA detect/quant	Negative	—	—	—	—
*Staphylococcus aureus* nasal polymerase chain reaction (PCR)	Positive	—	—	—	—
Methicillin‐resistant *Staphylococcus aureus*	Negative	—	—	—	—
*Mycoplasma pneumoniae* Ab	IgM negative IgG positive	—	—	—	—
*Chlamydia pneumoniae* Ab	IgM negative	—	—	—	—
*Chlamydia trachomatis* Ab	IgM and IgG negative	—	—	—	—
Chlamydia IgM and IgG panel	IgM and IgG negative	—	—	—	—
*Group A Streptococcus* PCR	—	—			Negative
*Adenovirus*	—	—	—	—	Negative
*Coronavirus* 229E	—	—	—	—	Negative
*Coronavirus* HKU1	—	—	—	—	Negative
*Coronavirus* NL63	—	—	—	—	Negative
*Coronavirus* OC43	—	—	—	—	Negative
*SARS Coronavirus* 2	—	—	—	—	Negative
*Human metapneumovirus*	—	—	—	—	Negative
*Human rhinovirus/enterovirus*	—	—	—	—	Negative
*Influenza A virus*	—	—	—	—	Negative
*Influenza B virus*	—	—	—	—	Negative
*Parainfluenza Virus* 1	—	—	—	—	Negative
*Parainfluenza Virus* 2	—	—	—	—	Negative
*Parainfluenza Virus* 3	—	—	—	—	Negative
*Parainfluenza Virus* 4	—	—	—	—	Negative
*Respiratory syncytial virus*	—	—	—	—	Negative
*Bordetella parapertussis*	—	—	—	—	Negative
*Bordetella pertussis*	—	—	—	—	Negative
*Chlamydia pneumoniae*	—	—	—	—	Negative
*Mycoplasma pneumoniae*	—	—	—	—	Negative
*Coccidioides Ab screen*	Negative	—	—	—	—
*Blastomyces Ab*	Negative	—	—	—	—
*Borrelia miyamotoi*, PCR	Negative	—	—	—	—
*Ehrlichia/Anaplasma* PCR	Negative	—	—	—	—
*Babesia*, PCR	Negative	—	—	—	—
*Aspergillus* Ab	Negative	—	—	—	—
Hepatitis Bs Ag screen	Nonreactive	—	—	—	—
Hepatitis B core IgM Ab	Nonreactive	—	—	—	—
Hepatitis A IgM Ab	Negative	—	—	—	—
HCV Ab w/reflex to HCV PCR	Negative	—	—	—	—
Human Immunodeficiency Virus 1/‐2 Ag and Ab screen	Negative	—	—	—	—
Broad range bacteria PCR + sequencing	—	Positive for *F. necrophorum*	—	—	—

After the blood cultures were obtained, the patient was initiated on broad‐spectrum intravenous (IV) antibiotics with vancomycin (1250 mg IV every 8 h), piperacillin–tazobactam (3.375 g IV every 6 h), and doxycycline (100 mg IV every 12 h) to cover for the most common bacterial causes of community‐acquired pneumonia (CAP) and for anaerobes given the necrotizing component. Despite this treatment, he continued to have persistent fever, tachycardia, and tachypnea, and he developed acute hypoxemic respiratory failure (PaO_2_/FiO_2_ = 250 mmHg) requiring supplemental oxygen at 5 L/min to keep oxygen saturation above 90%. Pneumonia severity index (PSI) was 97 (Risk Class IV—high). Once influenza was ruled out, methylprednisolone 40 mg IV daily for 5 days was added for severe CAP. Given this clinical worsening, repeat CT of the chest, abdomen, and pelvis was done, and it showed the development of new bilateral pleural effusions, concerning for empyema (Figure 3). After 48 h, blood culture in the anaerobic bottle grew *F. necrophorum.* Antimicrobial susceptibility showed sensitivity to clindamycin, metronidazole, and penicillin G (MIC ≤ 0.5 for PCN G, determined by CLSI M11 agar dilution), and at that point, vancomycin and doxycycline were discontinued. CT neck venogram was negative for septic thrombophlebitis of internal jugular veins and did not show any evidence of neck abscess or lymphadenopathy. Thoracentesis was performed and was suggestive of empyema (Table 2) with cloudy fluid and a total of 350 cc of pleural fluid drained. Anaerobic culture of pleural fluid remained without growth. Repeat CT chest, obtained 24 h post thoracentesis, demonstrated residual fluid collection in the left pleural space. A percutaneous pigtail catheter was placed in the left pleura with 6 doses of tPA (dornase). Repeat blood culture and pleural fluid cultures remained without growth.

Figure 3Contrast‐enhanced computed tomography (CT) of the chest in the (a) sagittal, (b) axial, and (c) coronal planes, demonstrating marked progression of pleural effusions, larger on the left (L > R), with left‐sided loculation and associated lung base atelectasis.

**Figure:**
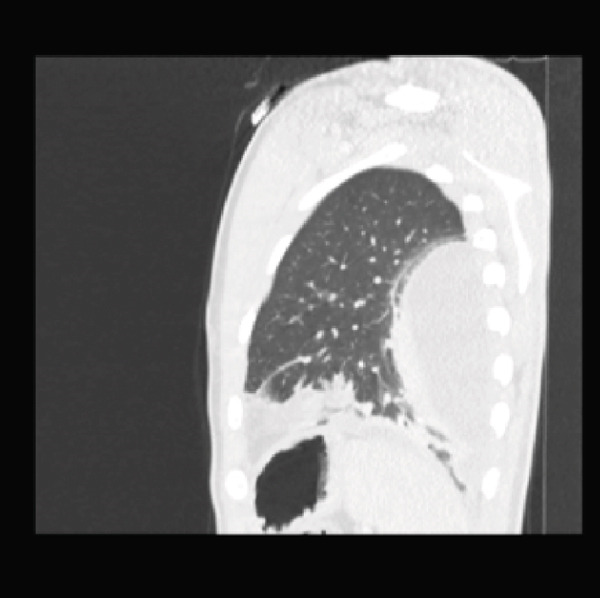
(a)

**Figure:**
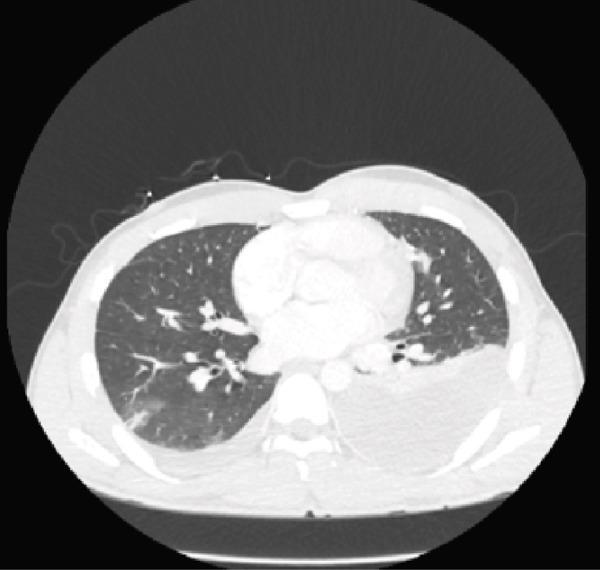
(b)

**Figure:**
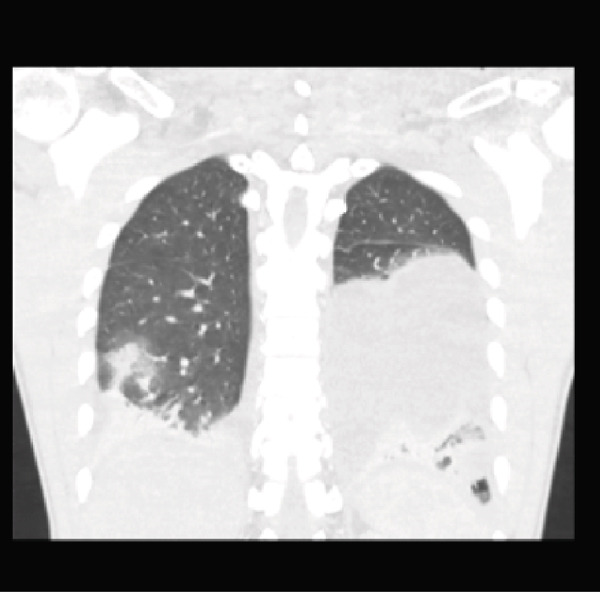
(c)

**Table 2:** This table represents pleural fluid analysis consistent with empyema.

	**Reference range**	**Admission**
PH (left pleural)	< 7.2	7.00 (left) 7.28 (R)
LD/serum LD (left pleural)	< 0.6	> 12.25 (L) 4.75 (R)
Pleural total protein/serum total protein (left pleural)	< 0.5	0.69 (L) 0.64 (R)
Pleural total nucleated cells (left pleural)	< 500	11,275 (L) 3568 (R)
Pleural gram stain (left pleural)	—	White blood cells
Cytology (right pleural)	—	Negative for malignancy

However, the patient developed up trending transaminitis with peak values on hospital day 5 (AST 151 [reference range 8–48 U/L], ALT 170 [reference range 7–55 U/L], ALP 217 [reference range 40–129], direct bilirubin 0.7 [reference range 0.0–0.3], indirect bilirubin 2.0 [reference range 0.0–1.2]) (Table 3). Work‐up for viral hepatitis; vascular, metabolic, and inherited liver diseases; and liver abscess was negative, and the patient was diagnosed with drug‐induced liver injury (DILI) due to piperacillin–tazobactam. At that point, piperacillin–tazobactam was discontinued and ampicillin–sulbactam monotherapy at the dose of 3 g IV every 6 h was started based on susceptibility results. Left‐sided pleural effusion continued to decrease, and the pigtail catheter was removed after 4 days. Unfortunately, the right‐sided pleural effusion continued to worsen. Repeated right thoracentesis was performed with 55 mL of pleural fluid drained, with results consistent with empyema, while pleural cytology was obtained and was negative for malignancy. Ultimately, the patient required an additional pigtail catheter in the right pleural space given thoracentesis findings and up trending inflammatory markers (Table 3). Surgical decortication for source control was considered but not performed, as the patient had improved, sepsis had resolved, the collections were decreasing with lung re‐expansion, and there was no evidence of a trapped lung. Both right and left pleural fluid polymerase chain reaction (PCR) sequencing also detected *F. necrophorum.* The patient was discharged with 4 weeks of amoxicillin–clavulanic acid therapy at the dose of 875–125 mg two times daily. Following 6 weeks of antibacterial therapy, his symptoms resolved, and follow‐up CT chest showed a complete resolution of the pleural effusions, although with residual pleural thickening.

**Table 3:** This table illustrates the trend of selected laboratory values, including complete blood cell count, liver enzymes, and inflammatory markers.

	**Reference range**	**Admission**	**Day 1**	**Day 3**	**Day 5**	**Day 7**	**Day 10**	**Day 15 discharge**	**Day 23 follow up**
Hemoglobin	13.2–16.6 g/dL	14.5	12.6	11.5	12.3	12.0	11.5	10.0	12.2
Platelet count	135–137 × 10(9)/L	91	79	115	289	436	634	950	462
White blood cell count	3.4–9.6 × 10(9)/L	19.2	14.1	23.6	18.4	14.8	17.2	12.0	8.2
Bilirubin, total	0.0–1.2 mg/dL	2.0	1.8	1.5	1.3	1.1	—	—	0.2
Alkaline phosphatase	40–129 U/L	199	150	197	153	127	—	—	217
Alanine aminotransferase (ALT)	7–55 U/L	48	40	52	170	127	—	—	104
Aspartate aminotransferase (AST)	8–48 U/L	36	33	105	108	50	—	—	48
C‐reactive protein	< 5.0 mg/L	195	170	154.0	63.8	79.2	—	—	—
Procalcitonin	0.0–0.24 ng/mL	14.9	—	3.90	1.17	—	—	—	—
D‐Dimer	≤ 500 ng/mL FEU	6020	4981	1894	5118	4569	—	—	—
Ferratin	31–409 mcg/L	741	—	685	715	1003	—	—	—

## 3. Discussion

First described by Andree Lemierre, a French bacteriologist, LS represents a form of postanginal septicemia manifesting as a triad of bacteremia, clinical and/or radiological evidence of internal jugular vein thrombophlebitis, and septic emboli developing during or after acute pharyngeal infection. Internal jugular vein septic thrombophlebitis typically manifests as tenderness and swelling of the sternocleidomastoid muscle and is a hallmark of LS in its typical form [16, 17]. While the primary causative agents of LS are anaerobic bacteria, most often *F. necrophorum and Fusobacterium nucleatum,* streptococci, staphylococci, and *Klebsiella pneumoniae* have been described as well [16, 18].

Variants of LS include cases of oropharyngeal infection with sepsis and evidence of embolic complications, but without internal jugular thrombosis. These are termed “incomplete LS” [19]. LS variants also include genitourinary and gastrointestinal variants, such as *F. necrophorum* pylephlebitis associated with liver abscess [20, 21] or mesenteric vein thrombosis associated with diverticulitis or appendicitis [22]. Given the rarity of LS variants, there is a delay in diagnosis and timely treatment, which might negatively impact the outcome and increase mortality. Although the term “incomplete LS” is used in the literature to describe *F. necrophorum* infections that do not meet all criteria for classic LS, our case could also be reported as *F. necrophorum* sepsis without jugular thrombophlebitis. We elected to retain the term incomplete LS to emphasize that *Fusobacterium* infections can present in atypical forms. Recognition of these variants is essential, as limited awareness may contribute to diagnostic delay.

*F. necrophorum* is a virulent pathogen capable of causing necrotizing infection and abscess formation, including necrotizing pneumonia, even in the absence of LS [15, 23]. In young, otherwise healthy adults, such as our patient, *F. necrophorum* induces severe pulmonary necrosis through the action of virulence factors like leukotoxin, which disrupts neutrophil function and promotes tissue destruction, leading to abscess formation and cavitation. In addition to leukotoxin, *F. necrophorum* expresses hemagglutinins, hemolysins, and outer membrane proteins that facilitate adhesion, tissue invasion, and immune evasion. The bacterium′s lipopolysaccharide (LPS) has potent endotoxic activity, contributing to local inflammation, vascular injury, and further tissue necrosis. The synergistic effects of these factors enable *F. necrophorum* to establish deep‐seated infection within the lung parenchyma, resulting in liquefactive necrosis and abscess formation [3, 24].

Our case is similar to few reported in the literature that introduced the term “incomplete LS” where patients had a rapidly progressive infection, complicated with pneumonia and empyema but lacked the hallmark sign of internal jugular vein thrombosis [18, 25]. These patients developed severe infections complicated with sepsis and septic shock. This is to highlight that *F. necrophorum* should not be ruled out based on the lack of demonstrated thrombosis, which was originally described as part of the syndrome. *F. necrophorum* can sometimes develop in the background of viral coinfection with *Epstein-Barr virus* (EBV) or *Cytomegalovirus* (CMV) [26]. Fatal cases of coinfection in young and healthy adults have been reported with *Legionella pneumophila* pneumonia and *F. necrophorum.* In this case, the cultures remained negative, but coinfection was diagnosed by using metagenomic next‐generation sequencing from bronchoalveolar lavage (BAL) and blood samples [27]. Coinfection with other pathogens such as *Streptococcus*, *Peptostreptococci*, *Bacteroides*, *Arcanobacterium*, and *Streptococcus* spp. has been reported as well [26].

Prior to hospitalization, our patient was seen in urgent care and received oral 40‐mg prednisone for 3 days. The impact of steroids alone (without antibiotics) on bacterial pneumonia progression is not well established. Observational data suggest that immunosuppression from corticosteroids, even for short periods, may increase the risk of more severe infection if appropriate antimicrobial therapy is delayed. Therefore, while a brief course of prednisone is unlikely to have a major effect in most immunocompetent hosts such as our case, the absence of antibiotics during active bacterial infection could have contributed to clinical worsening. In our patient, due to progression to severe CAP associated with hypoxemic respiratory failure, adjunctive corticosteroid therapy was initiated. Severe CAP criteria are defined by the need for intensive care unit (ICU) admission, requirement for invasive or noninvasive mechanical ventilation, high oxygen requirements (PaO_2_/FiO_2_ < 300), or high severity scores such as PSI Class IV/V or CURB‐65 ≥ 3. Corticosteroids are not recommended for nonsevere CAP or in the absence of these severe features. In the setting of septic shock, the Surviving Sepsis Campaign and the Society of Critical Care Medicine also recommend corticosteroids only if shock is refractory to adequate fluid resuscitation and vasopressor support [12, 28, 29] The efficacy and safety of corticosteroids in severe CAP have been established in recent randomized controlled trials and meta‐analyses, which demonstrate that corticosteroids reduce mortality, time to clinical stability, and length of hospital stay in severe CAP but not in nonsevere cases. Typical regimens include hydrocortisone 200 mg per day (either as a continuous infusion or divided doses) or methylprednisolone 40–80 mg per day, administered for 5–7 days. The most common adverse effect is hyperglycemia; importantly, no consistent increase in secondary infections or mortality has been observed in bacterial CAP [28, 30]. There are no randomized controlled trials or direct data regarding the use of corticosteroids specifically in *F. necrophorum* infections. All recommendations for corticosteroid use in this context are extrapolated from studies of severe CAP of mixed bacterial etiologies. The clinical criteria for corticosteroid initiation remain the same regardless of the underlying pathogen, including *F. necrophorum*.

In young and otherwise healthy patients such as the one we report, the leading differential diagnosis for community‐acquired cavitating pneumonia should include PVL‐positive *S. aureus*; aspiration pneumonia, which is typically polymicrobial and/or involves anaerobic pathogens; *Klebsiella*; septic pulmonary emboli from distant primary foci of infection; tuberculosis; and endemic fungal infections. In Wisconsin, these include blastomycosis, histoplasmosis, and coccidioidomycosis.

Necrotizing pneumonia diagnosis requires a high clinical suspicion due to the possibility of rapid decompensation even in younger and healthy individuals like our patient. Timely and accurate diagnosis is the key to initiating appropriate management measures including antibiotics and source control measures such as chest tube placement and thoracotomy. Due to the need for prolonged antibiotic treatment, maximum effort should be made to identify the causative pathogen [6]. If cultures are not obtained prior to the initiation of antibiotics, isolation of organisms could be further difficult as the growth of most common CAP pathogens is easily inhibited by a few doses of first‐line antibiotic therapy. The gold standard imaging modality for necrotizing pneumonia is CT chest with IV contrast as radiograph has a lower sensitivity [31, 32].

Sputum culture remains the gold standard of microbiological diagnosis. IDSA guidelines recommend sputum culture collection in all patients with severe pneumonia as well as patients with concern for methicillin‐resistant *Staphylococcus aureus* (MRSA) and *Pseudomonas aeruginosa* pneumonia. However, collection and yield can be unreliable, especially given that some common respiratory pathogens such as *Legionella pneumophilia* require specialized media [33]. Therefore, molecular diagnostic methods are being widely used with the advantages of shorter turnaround time and less impact due to antibiotic exposure. These include multiplex real‐time PCR assays, antigens (*Legionella* urinary antigen, *Streptococcus pneumoniae* urinary antigen, galactomannan), and serology. Studies have shown that the use of multibacterial and multiviral PCR panels has doubled pathogen detection in CAP from 39.3% to 86.7% [34]. Fibrotic bronchoscopy with BAL and mini‐BAL should be performed when spontaneous and induced sputum sample collection remains a challenge. Procalcitonin may be helpful in differentiating bacterial versus viral etiologies, but there is no verified cutoff value, and it can be affected by other variables such as renal function. Therefore, it should not be used as a sole marker for diagnosis or assessing clinical improvement. While IDSA guidelines do not recommend obtaining blood cultures in patients with nonsevere CAP, especially in the outpatient setting, blood cultures are recommended in in patients with severe CAP. In our case, blood cultures were the first to reveal *F. necrophorum* on hospital day 2, which was later confirmed by pleural fluid 16s PCR. The first step of necrotizing pneumonia management is risk factor‐based broad‐spectrum IV antibiotic therapy until pathogen detection. In patients with prior cultures positive for MRSA or *Pseudomonas aeruginosa* or patients with significant epidemiological risk factors for these organisms, broad‐spectrum antibiotics such as vancomycin and cefepime should be used. While anaerobes are common pathogens in lung abscesses due to odontogenic sources, their involvement in necrotizing pneumonia and pulmonary gangrene is not well established. Thus, anaerobic coverage may be considered if there is concern for an odontogenic source [35].

Up to 40% of patients with CAP admitted to the ICU will experience clinical deterioration after initial stabilization. Therefore, early identification of the pathogen and its antibiotic sensitivities is important to ensure appropriate antibiotic coverage [36]. There may be a role for adding antibiotics with antitoxin effects such as linezolid, clindamycin, and rifamycin when suspecting or treating toxin‐producing Gram‐positive organisms such as *S. aureus*, Group A *Streptococcus*, and invasive pneumococcal pneumonia [37]. Furthermore, the use of IVIG in severe necrotizing pneumonia was explored in the CIGMA study where a polyclonal antibody preparation (trimodulin) showed no significant differences in ventilator‐free days compared to placebo but showed improved mortality outcomes in post hoc analysis [38].

*F. necrophorum* is susceptible to penicillin, *β*‐lactam/*β*‐lactamase inhibitor combinations, carbapenems, and metronidazole. However, emerging resistance has been reported: 2%–5% of isolates show resistance to penicillin G [39]. Clindamycin and tetracycline resistance can occur, often associated with the presence of resistance genes such as tet (M), tet (32), erm (A), and erm (B), which correlate with elevated MICs [40]. Erythromycin resistance is notable, with up to 15% of isolates affected [41]. All isolates remain sensitive to *β*‐lactam/*β*‐lactamase inhibitors (e.g., amoxicillin/clavulanate), carbapenems, and metronidazole. Local susceptibility patterns should be considered due to emerging resistance, particularly to penicillin, clindamycin, and macrolides [38–40]. Our patient was initially treated with piperacillin–tazobactam; however, due to the development of DILI, antimicrobials were switched to ampicillin/sulbactam, given susceptibilities indicating penicillin minimum inhibitory concentration (MIC) 0.5. Ampicillin/sulbactam was continued until the patient was discharged on Day 15. At that time, amoxicillin/clavulanate 875–125 twice daily was started for an additional 3 weeks. He completed a total of 5 weeks of treatment.

Evaluating clinical progression and exploring source control options are the next steps, as often antibiotics alone are not sufficient given aggressive pathogens and the extent of disease. This helps avoid complications such as pleural involvement and progressive parenchymal destruction [8]. While daily chest x‐rays may be useful, a short interval CT scan might yield more valuable information, as outlined above. Fukushima et al. described a case of *Fusobacterium nucleatum* necrotizing pneumonia complicating *Influenza A*, who was successfully treated with ceftriaxone and azithromycin without the need for source control, while all five patients with necrotizing pneumonia described in Chatha et al.′s case series required chest tubes for source control, and three of them ultimately needed thoracic surgery [8]. Empyema should be drained with chest tube placement; our patient required bilateral chest tubes despite susceptibility‐based antibiotic treatment given clinical worsening with progressive pleural fluid accumulation [6].

Given highly variable clinical presentation and progression of necrotizing pneumonia, risk factors for developing complications requiring procedural intervention are not well understood [42]. In fact, Hoffer et al. noted a 100% failure rate and a 70% rate of bronchopleural fistula when percutaneous drainage was used to treat liquefying necrotizing pneumonia as opposed to a discrete, well‐defined abscess [43]. Pulmonary gangrene, differentiated from necrotizing pneumonia due to central vascular and bronchial obstruction as well as significant cavitation, on the other hand, requires surgical debridement of dead parenchyma through wedge resection, lobectomy or bilobectomy, and pneumonectomy [8, 42, 44]. Therefore, some of the indications for surgical resection of lung parenchyma in the setting of acute necrotizing lung infections include massive hemoptysis, pulmonary gangrene, or inadequate response to therapy. Our patient improved with chest tube drainage alone and did not develop gangrenous changes requiring surgical resection.

Point‐of‐care ultrasound (POCUS) is an emerging diagnostic tool that has the potential to help guide clinical decision‐making, especially in cases where timely diagnosis is crucial. Bedside ultrasound provides powerful insight into the presence of a pleural effusion with characteristics such as volume, echogenicity that help delineate transudative from exudative effusions, and the presence of septations that keys clinicians into the presence of a possible empyema [43–46]. Although CT imaging is often definitive for the assessment of pleural effusions, initial evaluation and subsequent resolution/follow‐up can be estimated by POCUS. As with any tool, there are limiting factors. For POCUS, this includes operator dependence with subjective interpretations and technical challenges including body habitus and the presence of artifacts such as bowel gas. Additionally, it has been shown to provide more sensitivity (94.54% vs. 67.68%) and specificity (97.88% vs. 85.30%) than chest radiographs [47]. Nevertheless, POCUS is not to replace clinical judgment or be used as a substitute for other diagnostic tools. It is instead a convenient supplemental window that can aid timely diagnosis and follow‐up of insidious presentations as with necrotizing pneumonia.

## 4. Clinical Pearls

• Consider *F. necrophorum* in adolescents and young adults who present with severe pharyngitis followed by sepsis or necrotizing pneumonia, even in the absence of internal jugular vein thrombosis.
• Preferred antimicrobial therapy for *F. necrophorum* includes a *β*‐lactam/*β*‐lactamase inhibitor or a carbapenem. Metronidazole may be added if the selected regimen does not provide adequate anaerobic coverage.
• Avoid clindamycin and macrolides due to the potential for antimicrobial resistance.
• Source control is essential in the management of empyema. Recommended measures include early drainage, consideration of tPA plus DNase for loculated collections, and reserving surgical intervention for cases of treatment failure or the presence of a trapped lung.

## Ethics Statement

The patient provided informed consent to participate in this case study. Ethical approval was not required for this type of study in accordance with institutional guidelines.

## Consent

Informed consent was obtained from the patient for publication of this case study and any accompanying images or data.

## Conflicts of Interest

The authors declare no conflicts of interest.

## Funding

No funding was received for this manuscript.

## Data Availability

The data that support the findings of this study are available from the corresponding author upon reasonable request.
